# Cooperative climate action under background risk

**DOI:** 10.1038/s41598-025-12340-9

**Published:** 2025-07-25

**Authors:** Hao Luo, Hanna de Boer, Oliver Musshoff, Daniel Hermann

**Affiliations:** 1 Department of Agricultural Economics and Rural Development, University of Göttingen, D-37073 Göttingen, Germany; 2 University of Bonn, D-53115 Bonn, Germany

**Keywords:** Climate change, Background risk, Public goods game, Laboratory experiment, Climate-change mitigation, Climate-change mitigation, Psychology and behaviour

## Abstract

Addressing climate change requires collective action, yet individuals face a collective-risk social dilemma: we must invest in mitigation efforts at a personal cost, with no guarantee that others will contribute, while the benefits are shared globally. Economic theory predicts that the presence of background risk, such as geopolitical instability, pandemics, and economic crises, makes individuals more cautious, potentially reducing their contributions to collective climate protection. As these uncertainties grow, concerns arise that exogenous risks may weaken climate cooperation at a time when it is most urgent. To test this prediction, we conducted an economic laboratory experiment with treatments incorporating background risk into a threshold Public Goods Game framed around climate protection, with real carbon offset purchases linked to participants’ decisions in the experiment. Contrary to theoretical expectations, we find that background risk does not systematically lower the likelihood of reaching collective climate goals. This suggests that, under conditions where climate risks are well-communicated and cooperation incentives are strong, concerns about background risk undermining climate action may be less pronounced than anticipated.

## Introduction

Despite decades of both international and national efforts, climate change continues to be a persistent challenge. In 2023, the global average surface temperature reached its highest level since 1880, rising $$1.45\,^{\circ }\text {C}$$ above pre-industrial levels^1^ and edging dangerously close to the $$1.5\,^{\circ }\text {C}$$ threshold set by the Paris Agreement. That same year, humanity surpassed six out of the nine planetary boundaries^2^, signaling growing pressure on Earth’s resilience and underscoring the fragility of our life-support systems. As climate change intensifies, the frequency of weather-, climate-, and water-related disasters has increased fivefold over the past 50 years, leading to over 2 million deaths and economic losses totaling US$3.64 trillion^3^. The 2023 Intergovernmental Panel on Climate Change (IPCC) report^4^ warns that exceeding $$1.5\,^{\circ }\text {C}$$ of warming could lead to irreversible and irreparable consequences for ecosystems and human societies. Human activities are the primary drivers of global warming, underscoring the urgent need for immediate and ambitious mitigation efforts. Yet, the window of opportunity to ensure a sustainable and livable future is narrowing rapidly.

Beyond environmental threats, individuals and societies are increasingly exposed to additional layers of risk. The COVID-19 pandemic, geopolitical conflicts, economic instability, trade tensions, and cyber threats collectively hinder progress toward global sustainability goals. As highlighted in the 2024 Sustainable Development Goals (SDG) progress report^1^, these growing uncertainties have substantially jeopardized efforts to meet the 2030 Agenda.

Many of these risks are non-diversifiable or not fully insurable, leaving individuals exposed to uncertainties beyond their control. Theoretical and empirical research suggests that individuals facing such background risk - exogenous, immutable risks that are beyond individual control and independent of endogenous choices - adjust their risk-taking behavior accordingly. Specifically, the presence of background risk tends to make risk-averse individuals behave in a more risk-averse way toward any other independent risks, a phenomenon known as risk vulnerability^5,6^. Background risk influences decision-making in various economic contexts, from insurance demand, investment decisions to risk sharing mechanisms^7–9^. In an interconnected world, decisions about climate protection do not occur in isolation but rather under the simultaneous presence of these independent risks.

Climate protection represents the largest public goods challenge, representing a collective-risk social dilemma. Individuals must invest in mitigation at their own expense, with benefits extending to all, yet without any guarantee that others will contribute. Unlike other social dilemmas, climate action requires individuals to make repeated decisions before the outcome is evident, involves non-refundable investment costs, carries uncertainty about the actual value of preventing dangerous climate change, and puts remaining private resources at risk if the target goal is not reached^10^. These features have led some to describe it as a “gamble for the climate.”^11^ Given this element of “gambling,” individuals’ willingness to cooperate may be influenced by their exposure to independent background risk. Within the risk vulnerability framework, individuals accounting for such independent risks in their risk-taking decisions tend to behave more cautiously and may prioritize personal security, which in turn reduces their contributions to collective efforts. Studies that isolate climate-related risks without accounting for background risk may overestimate cooperation levels, limiting their real-world applicability.

In this study, we experimentally examine the theoretical prediction concerning individual cooperative behavior in climate protection under background risk. Specifically, we conducted an economic laboratory experiment using a modified threshold Public Goods Game (PGG) based on the framework proposed by Milinski et al. (2008)^10^. Threshold PGG has been extensively studied both theoretically and experimentally^12–14^. In a typical threshold PGG, each player in a group receives an endowment and decides how much to contribute to a public good. If the aggregate group contribution meets or exceeds a certain threshold, the public good is provided. Milinski et al. (2008)^10^ adapted this framework to better reflect the nature of climate change, where contributions are made not to achieve a gain but to prevent a loss. In their design, failure to reach the target sum results in a risk of losing the remaining personal endowment. They find that only under a high risk (90%) of simulated dangerous climate change do half of the groups reach the target, concluding that one way to address the collective-risk social dilemma of climate change is to emphasize that insufficient investment is likely to result in serious financial loss to the individual. Subsequent experiments have expanded this design to provide a more realistic perspective, incorporating factors such as inequality and communication^15^, time horizons and intermediate goals^16^, intra- and intergenerational discounting^17^, threshold uncertainty^18^, wealth and loss heterogeneities^19^, and polycentric governance with subgroup cooperation^20^. While these studies have effectively captured various real-world challenges relevant to climate change cooperation, they overlook background risk, which can heighten risk-vulnerable behavior and reduce contributions to climate protection.

A central goal of this study is to reproduce real-world complex decision-making environments, where individuals navigate multiple risks simultaneously. In a globalized world, background risk is becoming increasingly prominent. Understanding specific risk-taking behavior requires accounting for the multiple risks individuals face in the background. This study addresses this gap by incorporating independent background risk into the analysis of climate cooperation. Climate cooperation, in particular, can be viewed as a form of risk-taking, as individuals must decide whether to contribute without certainty that others will do the same and without guaranteed returns on their mitigation efforts. By integrating background risk into an experimental setting, we offer a more realistic representation of climate cooperation within a multi-risk framework. From a theoretical perspective, background risk induces more cautious behavior among risk-averse individuals, potentially reducing their willingness to contribute to collective climate action. Recognizing this decline in climate cooperation is particularly relevant given current societal developments, where we are at a critical juncture for implementing effective climate mitigation efforts, yet increasing risks in the background may hinder collective action. In this context, acknowledging the role of background risk can help policymakers develop more effective incentives and institutional frameworks that foster sustained climate protection cooperation.

## Methods

### Experimental design

The basic design is based on a 10-period threshold PGG^10^. Subjects are randomly assigned to one of three conditions and placed in groups of six, interacting with the same group members throughout all periods. The identities of participants remain private, and they are referred to only as “Player *i*” ($i = 1, 2, ..., 6$) during the experiment. No communication among participants is permitted.

In the Control condition (*C*), background risk is not present. At the beginning of the PGG, each subject is endowed with $E_i = 40$ tokens (experimental currency units), which can be allocated between a private account and a public climate fund. The group’s collective goal is to contribute at least 120 tokens to the climate fund by the end of 10 periods. In period *t* ($t = 1, 2, ..., 10$), a subject *i* chooses an action from the set $c_i^t = \{0, 2, 4\}$, where her total contribution over all periods is $C_i = \sum _{t=1}^{10} c_i^t$ and the total contribution of this group is therefore defined as $\sum _{i=1}^{6} C_i$. If a group meets the 120-token target, subject *i* in that group can keep her remaining private endowments $R_i$ ($= 40 - C_i$); if the target is not reached, she loses $\alpha$ of her remaining endowments ($\alpha \cdot R_i$). Unlike most previous studies that impose a $\alpha$ probability of losing all remaining savings, we apply a $\alpha$ loss rate to minimize additional risks beyond the study’s primarily focused background risk and keep the complexity low. In the experiment, we set $\alpha = 90\%$. After each period, subjects are informed about the contributions of other participants and the group’s total contributions from the previous period. Additionally, they are shown cumulative contributions of both the individual and the group across all periods.

In Treatment 1 (*T*1), the conditions are identical to those in *C*, except for the introduction of a 20% risk. Specifically, regardless of whether the collective goal of 120 tokens is met, each subject faces a 20% probability of losing all her remaining endowments. After 10 periods, once the group’s success in avoiding climate damage is known, the computer randomly generates a uniformly distributed number between zero and one for each group. If the number is less than or equal to 0.8, group members keep their remaining private endowments; otherwise, they lose them entirely.

However, the introduction of a background risk may reduce participants’ monetary incentives to contribute. Table 1 displays the expected payoffs for cases in which the threshold is reached and not reached, respectively, across all control and treatment conditions. In *C*, the difference in subject *i*’s expected payoff between reaching and not reaching the threshold is $\alpha \cdot (40 - C_i)$, whereas in *T*1, it decreases to $0.8 \cdot \alpha \cdot (40 - C_i)$. To account for the variation in incentives, we introduce a multiplier of 1.25 in *T*2, ensuring that expected payoffs are equivalent to those in *C*. In *T*2, after the group’s success in avoiding climate damage is announced, private remaining endowments are first multiplied by 1.25. Then, a randomly drawn number determines the subject *i*’s final payoff in the threshold PGG.

Tokens allocated to the climate fund in all control and treatment conditions will be used to purchase EU CO_2_ emission certificates, with the process transparently communicated to participants. The experimental instructions provide an explanation of the EU CO_2_ emission certificates and inform participants where they can access the certificate purchase announcement later. If a group reaches the 120-token threshold, the entire climate fund will be used for certificate purchases; otherwise, only 50% of the fund will be used, following a similar design applied by Tavoni et al. (2011)^15^. Thus, we introduce a specific field context where participants’ decisions in the experiment contribute to real-life climate protection, making the task more realistic. Certificates were purchased after the experiment through the non-profit organization Compensators e.V.^21^. In total, we spent 1,575.75€ on emission certificates, corresponding to 16.61 tons of CO_2_ at a price of 94.88€ per ton.

**Table 1:** Expected payoffs and incentive differences across experimental conditions. *Notes*: The table presents expected payoffs for cases where the threshold is reached and not reached, respectively. *Incentive difference* is computed as the absolute difference between the expected payoffs in these two scenarios.

Condition	Expected payoffs	Incentive difference
Control	$$\pi _i ={\left\{ \begin{array}{ll}40 - C_i & \hspace{6.3em} \text {if } \sum\limits _{i=1}^{6} C_i \ge 120 \\ (1 - \alpha ) \cdot (40 - C_i) & \hspace{6.3em} \text {if } \sum\limits _{i=1}^{6} C_i < 120\end{array}\right. }$$	$\alpha \cdot (40 - C_i)$
Treatment 1	$$\pi _i ={\left\{ \begin{array}{ll}80\% \cdot (40 - C_i) & \hspace{3.1em} \text {if } \sum\limits _{i=1}^{6} C_i \ge 120 \\ 80\% \cdot ((1 - \alpha ) \cdot (40 - C_i)) & \hspace{3.1em} \text {if } \sum\limits _{i=1}^{6} C_i < 120\end{array}\right. }$$	$0.8 \cdot \alpha \cdot (40 - C_i)$
Treatment 2	$$\pi _i ={\left\{ \begin{array}{ll}80\% \cdot (1.25 \cdot (40 - C_i)) & \text {if } \sum\limits _{i=1}^{6} C_i \ge 120 \\ 80\% \cdot (1.25 \cdot ((1 - \alpha ) \cdot (40 - C_i))) & \text {if } \sum\limits _{i=1}^{6} C_i < 120\end{array}\right. }$$	$\alpha \cdot (40 - C_i)$

### Experimental procedures

This study was ethically approved by the German Association for Experimental Economic Research e.V. (Institutional Review Board Certificate No. ijxYGbtI; accessible at https://gfew.de/ethik/ijxYGbtI). The experiment adhered to all relevant ethical guidelines and regulations, and informed consent was obtained from all participants. The study was pre-registered on AsPredicted (https://aspredicted.org/wm92-dwbh.pdf).

Subjects were recruited via hroot (Hamburg Registration and Organization Online Tool)^22^ and participated at either the BonnEconLab or the MPI Decision Lab in Bonn, Germany. Participants were primarily graduate and undergraduate students from Bonn and its surrounding areas. A total of 324 subjects (48.46% female), including 12 participants in a pilot session, took part in the experiment between November and December 2024. Each session involved 12–18 participants interacting anonymously via computer using Z-tree^23^. The following analyses focus on 312 subjects from the main sessions, as the pilot session had a higher show-up fee.

The experiment consists of three components: a 10-period threshold PGG and a one-shot Trust Game (TG), presented in randomized order, followed by an ex-post questionnaire. While the TG is part of the experiment, its results are not the subject of this paper. Tests for order effects show no statistically significant differences in first-period or cumulative contributions between subjects who play the TG first and those who play the PGG first (Mann–Whitney–Wilcoxon rank-sum test, $p = 0.120$ and $p = 0.847$, respectively). The questionnaire collects data on age, gender, study area, religion, political orientation, income level, Big Five personality traits, risk attitudes, and beliefs about climate change. Personality traits are measured using the BFI-10^24^, a short version of the Big Five Inventory that captures openness, conscientiousness, extraversion, agreeableness, and neuroticism with two items per trait. Risk attitudes are elicited by the incentivized Multiple Price List (MPL) task^25^, where participants choose between a safer option (low variance) and a riskier option (high variance) across ten paired lottery choices. The switch point from the safer option to the riskier option is used to identify individual risk attitudes : the later the switch, the more risk-averse the individual. Climate change beliefs are assessed using a set of statements evaluating participants’ perceptions on its occurrence and underlying causes, ranging from human activities to natural factors^26^. We constructed an index ranging from one to four, where one indicates the belief that climate change is mainly caused by human activities, and four reflects belief that there is insufficient evidence or that climate change is not occurring. Note 1 provides the experimental instructions for all control and treatment conditions.

Each session lasted approximately 55 minutes, with the PGG and TG each taking around 17 minutes. The remaining time was used for the instructions, a questionnaire, and final payout procedures. Payoffs are converted at a rate of 1 token = 0.25€ at the end of the experiment. The average total earnings were 20.24€, consisting of 4.33€ from the PGG, 3.57€ from the TG, and 2.34€ from the MPL task, plus a guaranteed 10€ show-up fee. Payments are made in cash at the end of each session.

### Predictions

We consider a utility function that satisfies standard properties: it is twice partially differentiable, strictly monotone increasing, and quasi-concave. In *C*, the expected utility of subject *i* can be expressed as:

1 $$\begin{aligned} EU_i^C = {\left\{ \begin{array}{ll} (40 - C_i)^{1-r} & \text {if } \sum\limits _{i=1}^{6} C_i \ge 120 \\ ((1 - \alpha ) \cdot (40 - C_i))^{1-r} & \text {if } \sum\limits _{i=1}^{6} C_i < 120 \end{array}\right. } \end{aligned}$$

where *r* denotes individual (constant) relative risk aversion coefficient, with $r = 0$ representing risk neutrality, $r > 0$ indicating risk aversion and $r < 0$ corresponding to risk-loving attitudes. The marginal expected utility of subject *i* is therefore given by:

2 $$\begin{aligned} \frac{dEU_i^C}{d(40 - C_i)} = {\left\{ \begin{array}{ll} (1 - r) \cdot (40 - C_i)^{-r} & \text {if } \sum\limits _{i=1}^{6} C_i \ge 120 \\ (1 - r) \cdot ((1 - \alpha ) \cdot (40 - C_i))^{-r} & \text {if } \sum\limits _{i=1}^{6} C_i < 120 \end{array}\right. } \end{aligned}$$

The relative difference in marginal expected utility between reaching and not reaching the threshold is formulated as:

3 $$\begin{aligned} \begin{aligned} \Delta \frac{dEU_i^C}{d(40 - C_i)} = \frac{ (1 - r) \cdot (40 - c_i)^{-r}}{(1 - r) \cdot ((1 - \alpha ) \cdot (40 - c_i))^{-r}} - 1 = \frac{1}{(1 - \alpha )^{-r}} - 1 \end{aligned} \end{aligned}$$

Equation 3 shows that the difference in subject *i*’s marginal expected utility between reaching and not reaching the threshold depends on her risk attitude parameter *r* and the exogenously given loss rate $\alpha$, assuming all else remains constant. As the loss rate increases, $1 - \alpha$ decreases, resulting in a larger marginal utility difference. Given the high loss rate in our study ($\alpha = 90\%$), we expect groups to exhibit higher provision rates in *C* compared to situations with lower loss rates.

When background risk is introduced (in *T*1 and *T*2), all subjects face an additional probability of losing their remaining private endowments, independent of whether the threshold is met. Assuming expected-utility preferences and risk-averse agents, adding a background risk to wealth makes individuals behave in a more risk-averse way with respect to any other independent risk^5,6^. Additionally, all commonly used Bernoulli utility functions that satisfy non-increasing harmonic absolute risk aversion exhibit risk vulnerability. This theory is empirically evident in different contexts that in the presence of background risk, subjects reduce the demand in risky assets^6,8,27–29^. One key feature of our experimental setup is that $E_i \ll 120$, meaning no single subject can reach the threshold alone and has no guarantee that others will contribute. Additionally, the no money-back guarantee further increases the risk associated with contributing to the public fund. Taken together, these factors make contributions to the climate fund a form of risk-taking behavior. Under the framework of risk vulnerability, subjects behave in a more cautious way after the introduction of background risk, shifting the marginal utility difference curve downward for risk-averse individuals. Therefore, the marginal utility differences between reaching and not reaching the threshold are smaller in *T*1 and *T*2 compared to *C*, leading us to the following hypothesis:

H1: The introduction of background risk reduces individual contributions to a public climate fund, resulting in fewer groups reaching the threshold.

Additionally, since Equation 3 holds for all control and treatment conditions, we further hypothesize:

H2: Individual risk attitudes influence their contributions to the public climate fund.

## Results

### Group contributions

Table 2 presents the aggregate group contributions and the proportion of groups that successfully reached the 120-token threshold across treatment conditions. As expected, aggregate contributions to the public good in *C* are high, with an average of 121.88 tokens. However, there is no substantial difference in group contributions between *C* and two treatment conditions, with average contributions in all three conditions slightly exceeding the 120-token threshold. Notably, the standard deviation is highest in *T*1, where participants face background risk and potential incentive effects, indicating greater variability in contributions within this treatment. A Kruskal–Wallis test indicates a statistically significant difference in contributions across three conditions ($p = 0.089$). Pairwise Mann–Whitney–Wilcoxon rank-sum tests reveal that contributions in *T*1 differ significantly from both *C* ($p = 0.069$) and *T*2 ($p = 0.051$). However, the difference between *C* and *T*2 is not statistically significant ($p = 0.862$). While the statistical tests indicate significant differences, the effect sizes are minimal, with variations only at the decimal level. Given the discrete nature of the action set, $c_i^t = \{0, 2, 4\}$, and the structure of the game, where the primary objective is to reach the threshold, these differences do not translate into meaningful economic implications. Therefore, we do not place particular emphasis on these results and instead focus on the success rates in achieving the threshold.

**Table 2:** Summary statistics of group contributions and the proportion of groups reaching the threshold. *Notes*: The unit of observation is the group.

**Treatment**	**Obs**	**Group contributions**				**Success rate**		
		**Mean**	**SD**	**Min**	**Max**	**Mean**	**Min**	**Max**
Control	17	121.882	4.152	116	132	0.882	0	1
Treatment 1	17	121.529	14.310	68	132	0.882	0	1
Treatment 2	18	121.000	8.657	92	140	0.944	0	1

Overall, there are no statistically significant differences in the proportion of groups that successfully reached the 120-token threshold across conditions ($p = 0.774$, Kruskal–Wallis test). In both *C* and *T*1, 15 out of 17 groups (88.2%) meet the threshold, with six groups in *C* and two groups in *T*1 contributing exactly 120 tokens. In *T*2, the success rate is slightly higher, with 17 out of 18 groups (94.4%) reaching the threshold, and seven groups contributing exactly 120 tokens. However, these differences are not statistically significant ($p = 0.512$ for both *C* vs. *T*2 and *T*1 vs. *T*2, chi-squared test). These results indicate that background risk does not systematically reduce the likelihood of reaching the threshold for climate protection, providing no empirical support for H1.

**Fig. 1:**
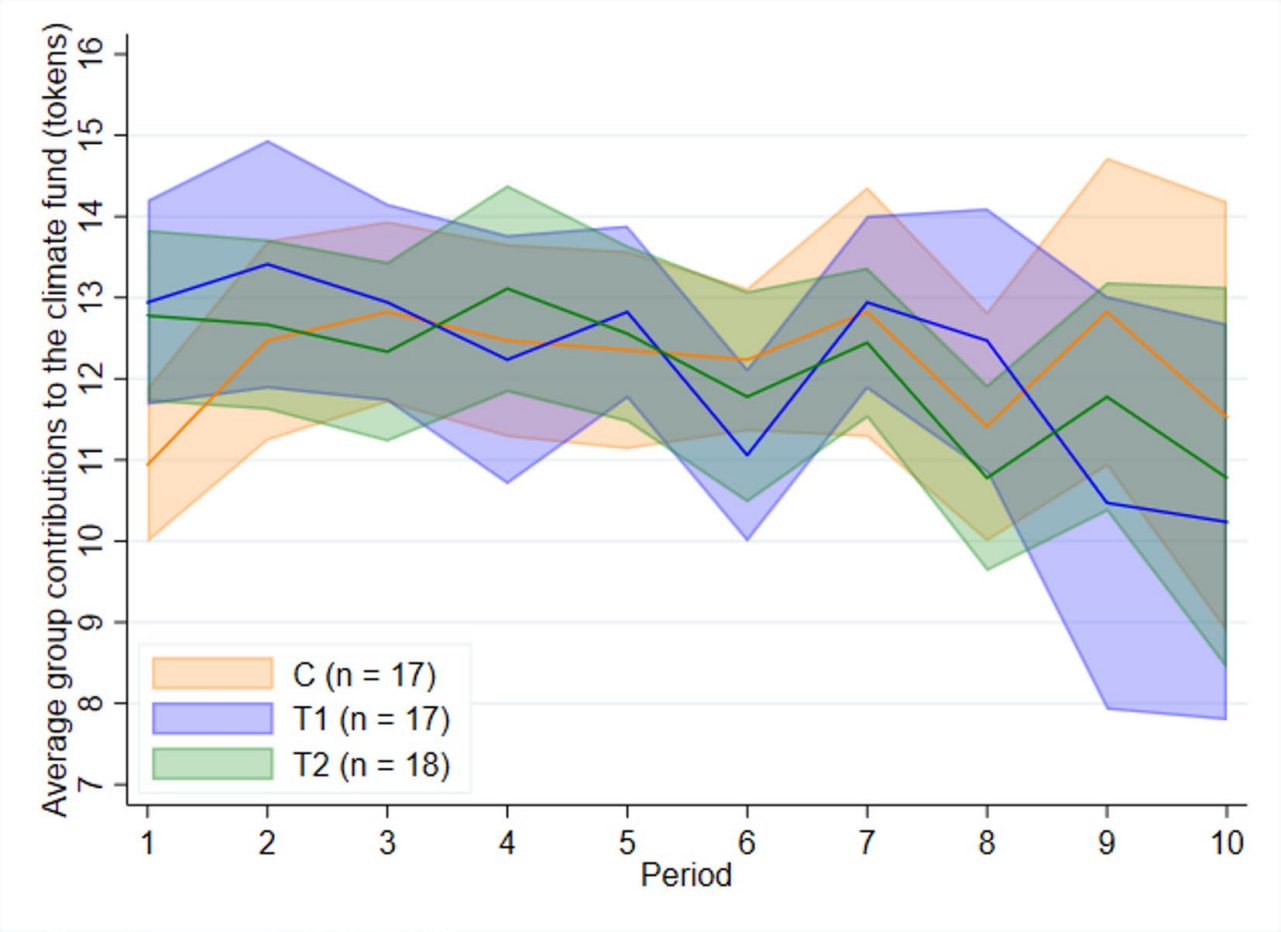
Average per-period group contributions over time. *Notes*: Shaded areas indicate 95% confidence intervals.

Fig. 1 illustrates the average per-period group contributions over time. Contributions in *C* remain relatively stable with lower variability, whereas *T*1 and *T*2 exhibit greater fluctuations (see Table 2) and a slight downward trend over time. However, the differences in standard deviations across treatments are not statistically significant ($p > 0.1$ for all pairwise comparisons, Levene test). An important characteristic of the collective-risk social dilemma is that contributions occur sequentially over multiple periods. As individuals gain experience, they may update their beliefs accordingly. Therefore, decisions made in the first period offer insights into a subject’s unconditional willingness to contribute, as they are made without any feedback on the actions of other group members. Group contributions in period 1 are higher in *T*1 and *T*2 (12.94 and 12.78 tokens, respectively) compared to 10.94 tokens in *C*. These differences are statistically significant at the 5% level ($p = 0.033$ for *C* vs. *T*1 and $p = 0.021$ for *C* vs. *T*2, Mann–Whitney–Wilcoxon rank-sum test).

### Individual contributions

Thus far, the analysis has focused on group-level contributions. In the following, we shift to the individual level to examine how individual characteristics influence contributions, conditional on treatments.

**Fig. 2:**
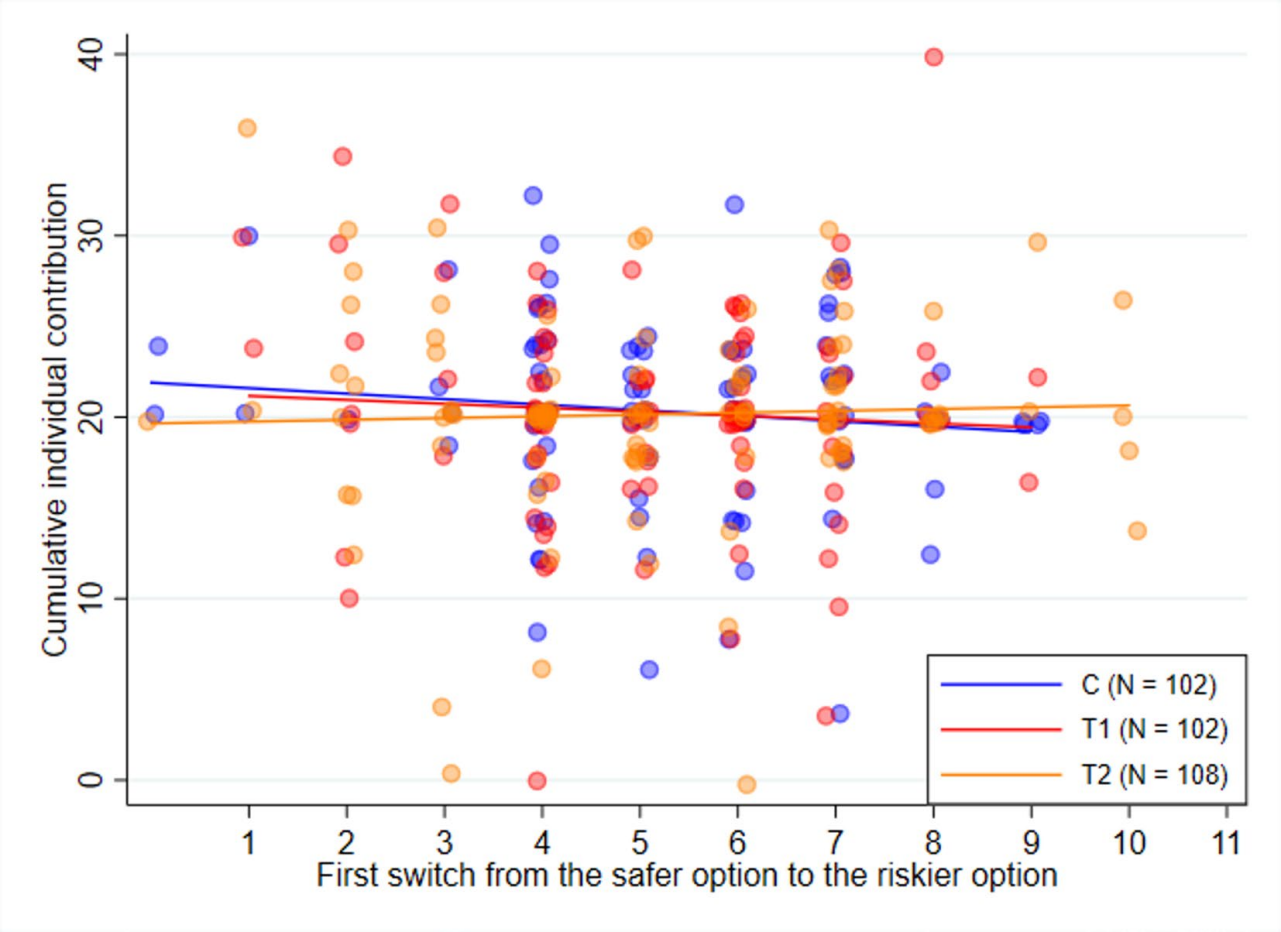
Relationship between individual risk attitudes and total contributions. *Notes*: The x-axis represents risk attitudes based on the first switch point in the MPL task^25^, where higher values indicate greater risk aversion. Each dot represents an individual observation, with fitted trend lines displayed for each treatment condition.

Based on theoretical considerations, individual risk attitudes can influence contributions to the public climate fund. The average switch point in the MPL task is 5.224, indicating that individuals are, on average, risk-averse. The majority of the subjects (61.54%) chose more than 4 safe choices, which is the predicted switch point of a risk-neutral subject. Kolmogorov–Smirnov tests show no significant differences in the distribution of risk attitudes across experimental conditions (all $p >= 0.200$). Fig. 2 displays the relationship between individual risk attitudes and cumulative contributions over all 10 periods. The fitted lines for *C* and *T*1 exhibit a slight downward slope, indicating a negative relationship between risk aversion and cumulative individual contributions. The regression line for *T*2 remains relatively flat, suggesting a negligible correlation. Overall, we do not find a statistically significant relationship between risk attitudes and individual contributions to the public climate fund, providing no empirical support for H2.

Table 3 presents the results of linear regressions with two dependent variables: cumulative individual contribution over all periods and individual contribution in period 1 to the public climate fund, controlling for treatment conditions and individual characteristics. Columns (1)–(4) report the results from Ordinary Least Squares (OLS) regression estimates, while Columns (5)–(8) present results from the ordered logit regression as robustness checks. To identify the monetary incentive effects, Columns (2), (4), (6), and (8) exclude data from *C*. The reference category for treatment dummies is *C* in Columns (1), (3), (5), and (7), and *T*1 in Columns (2), (4), (6), and (8). The results qualify the relationship identified at the group level, showing that first-period contributions are statistically significantly higher in both treatments than in *C* (Columns (3) and (7)). However, no statistically significant differences in cumulative individual contributions are observed across conditions (Columns (1) and (5)), offering no empirical support for H1. Additionally, the differences in both cumulative contributions and first-period contributions between *T*1 and *T*2 are negligible and statistically insignificant (Columns (2), (4), (6), and (8)), suggesting that variations in monetary incentives due to differing expected payoffs do not influence individual contributions in this experiment.

Considering individual characteristics, age, gender, and Big Five personality traits are three dimensions that statistically significantly influence contribution decisions. Cumulative individual contributions decline with age, as younger participants contribute statistically significantly more than older ones. Gender effects are observed in both cumulative individual contributions and first-period contributions, with females contributing more. Extraversion is associated with lower contributions, while agreeableness is positively related to contributions. As shown earlier, risk attitudes do not have a statistically significant effect on individual contributions to the climate fund. Although the climate change belief index is negatively correlated with contributions, namely individuals who strongly believe that climate change is occurring and primarily driven by human activity tend to contribute more, this relationship is not statistically significant.

**Table 3:** Linear regression of individual contributions. *Notes*: Columns (1)–(4) report the results from OLS regression estimates with robust standard errors, while Columns (5)–(8) present results from the ordered logit regression. Columns (2), (4), (6), and (8) exclude data from the Control condition. The reference category for treatment dummies is Control in Columns (1), (3), (5), and (7), and Treatment 1 in Columns (2), (4), (6), and (8). *N* represents the number of individuals. *, **, and *** indicate statistical significance at the 0.10, 0.05, and 0.01 level, respectively. Standard errors are shown in parentheses.

	(1)	(2)	(3)	(4)	(5)	(6)	(7)	(8)
	Cumulative contribution	Cumulative contribution	Contribution in period 1	Contribution in period 1	Cumulative contribution	Cumulative contribution	Contribution in period 1	Contribution in period 1
Treatment dummies								
T1	-0.617		0.260$$^*$$		-0.244		0.716$$^{**}$$	
	(0.766)		(0.136)		(0.258)		(0.353)	
T2	-0.241	0.390	0.300$$^{**}$$	0.035	-0.216	0.011	0.792$$^{**}$$	0.085
	(0.736)	(0.771)	(0.130)	(0.136)	(0.248)	(0.258)	(0.343)	(0.348)
Age (in years)	-0.199$$^{**}$$	-0.216$$^*$$	-0.013	-0.002	-0.055$$^{**}$$	-0.047	-0.032	-0.005
	(0.078)	(0.117)	(0.013)	(0.018)	(0.024)	(0.032)	(0.031)	(0.041)
Female	1.464$$^{**}$$	1.751$$^*$$	0.251$$^{**}$$	0.273$$^*$$	0.409$$^*$$	0.501$$^*$$	0.664$$^{**}$$	0.744$$^*$$
	(0.723)	(0.962)	(0.116)	(0.146)	(0.239)	(0.303)	(0.317)	(0.400)
Major in economics	-0.558	-0.905	-0.040	-0.041	-0.157	-0.261	-0.095	-0.096
	(0.738)	(0.955)	(0.128)	(0.157)	(0.238)	(0.300)	(0.320)	(0.415)
Having a religion	-0.436	-0.863	-0.133	-0.206	-0.071	-0.181	-0.338	-0.554
	(0.699)	(0.912)	(0.120)	(0.144)	(0.224)	(0.280)	(0.296)	(0.374)
Political orientation	-0.080	0.060	-0.013	0.010	-0.055	0.024	-0.030	0.023
	(0.184)	(0.239)	(0.031)	(0.035)	(0.062)	(0.077)	(0.082)	(0.103)
Income	0.095	0.280	-0.026	0.011	-0.012	0.053	-0.071	0.030
	(0.216)	(0.276)	(0.033)	(0.038)	(0.062)	(0.074)	(0.079)	(0.095)
Risk aversion	-0.015	0.095	-0.003	-0.000	-0.021	0.009	-0.009	-0.000
	(0.170)	(0.230)	(0.028)	(0.037)	(0.055)	(0.069)	(0.073)	(0.091)
Climate change beliefs	-0.690	-0.884	0.003	-0.024	-0.252	-0.364	-0.002	-0.061
	(0.555)	(0.791)	(0.089)	(0.074)	(0.202)	(0.269)	(0.267)	(0.360)
Extraversion	-0.812$$^{**}$$	-0.348	-0.172$$^{***}$$	-0.127$$^*$$	-0.263$$^{**}$$	-0.095	-0.454$$^{***}$$	-0.344$$^*$$
	(0.370)	(0.457)	(0.058)	(0.076)	(0.113)	(0.140)	(0.147)	(0.185)
Agreeableness	0.857$$^{**}$$	1.007$$^{**}$$	0.124$$^*$$	0.174$$^{**}$$	0.367$$^{***}$$	0.396$$^{**}$$	0.316$$^*$$	0.458$$^{**}$$
	(0.396)	(0.451)	(0.066)	(0.072)	(0.135)	(0.156)	(0.176)	(0.208)
Conscientiousness	0.019	-0.440	-0.095	-0.099	0.019	-0.138	-0.233	-0.252
	(0.344)	(0.483)	(0.058)	(0.079)	(0.120)	(0.164)	(0.161)	(0.218)
Neuroticism	0.097	0.002	-0.048	-0.016	0.037	0.009	-0.115	-0.040
	(0.364)	(0.481)	(0.052)	(0.066)	(0.118)	(0.146)	(0.149)	(0.181)
Openness	0.177	0.073	-0.060	-0.052	0.130	0.105	-0.143	-0.132
	(0.340)	(0.425)	(0.055)	(0.063)	(0.103)	(0.124)	(0.137)	(0.167)
Constant	24.342$$^{***}$$	22.439$$^{***}$$	3.229$$^{***}$$	2.518$$^{***}$$				
	(3.525)	(4.252)	(0.581)	(0.616)				
Observations	312	210	312	210	312	210	312	210

## Discussion

As highlighted by the 2023 IPCC report^4^, the window for effective climate action is rapidly closing, and human decisions remain the key determinant in limiting global warming. Climate action presents a global collective-risk social dilemma in which no single actor can ensure success independently, yet contributions from others are not guaranteed. At the same time, the returns on investments in climate resistance efforts are uncertain and often materialize only over time. If mitigation efforts fail to meet the necessary threshold, individual private resources are at risk. This inherent uncertainty creates a setting where individuals may “gamble” on climate protection. Importantly, assessing risk-taking attitudes toward climate protection in isolation may introduce bias, as real-world decisions are shaped by multiple, concurrent risks. Pandemics, wars, civil unrest, and political and societal divisions contribute to an environment where societies and individuals are increasingly exposed to background risks that are neither fully insurable nor diversifiable. To better understand individuals’ willingness to contribute to collective climate action, it is essential to account for the presence of independent background risk. The concept of risk vulnerability suggests that risk-averse individuals tend to take fewer risks when exposed to independent exogenous risks. Accordingly, our theoretical analysis predicts that under background risk, individuals will contribute less to public goods.

In this study, we empirically examine how individual willingness to contribute to climate mitigation efforts changes in the presence of background risk. We conducted a laboratory experiment incorporating a 20% independent exogenous risk into a threshold PGG framed around climate protection, where contributions to the public fund in the experiment are allocated to real-world CO_2_ emission reductions. Our experimental results do not provide evidence that background risk systematically reduces cooperation. At the aggregate group level, the presence of background risk does not statistically significantly affect the likelihood that a group reaches the contribution threshold. While we do not find a statistically significant effect of background risk on cumulative individual contributions, we observe an increase in first-period contributions. The higher provision rate in the first period in treatments contrasts with the theoretical prediction. It is important to note that our prediction is quite restrictive, as it ignores the timing of contributions and does not allow for statements about contribution behavior in each period. Signaling a willingness to contribute to the public good early on may be critical for the success of the group^30^. Empirical evidence suggests that successful groups tend to contribute more in the initial periods^15^, and high early contributions are positively correlated with subsequent cooperation^18^. The higher provision rate observed in the first period when background risk is introduced may have partially contributed to the high success rates observed in these treatments. Additionally, demographic and personality traits appear to influence contribution behavior. Cumulative individual contribution levels decrease with age, and females contribute more . Personality traits such as extraversion and agreeableness are associated with different levels of contribution as well. However, contrary to the prediction, risk attitudes do not have a statistically significant effect on cooperative behavior in climate protection. Surprisingly, climate change beliefs are also not significantly correlated with contributions. Notably, climate change beliefs in our sample are relatively homogeneous, with approximately 87% of students strongly believing that climate change is occurring and is primarily driven by human activity.

When interpreting these findings, it is important to consider the specific parameter choices of the experimental design. In the experiment, the loss rate is set high (at 90%), reflecting assessments of the 2023 IPCC report^4^ that many climate-related hazards, such as extreme heat, heavy precipitation, sea level rise, are *very likely* (90–100% probability) to intensify under further warming, with associated losses and damages to nature and people evaluated with *high confidence*. However, this high-loss setting represents a relatively favorable coordination environment and high success rates are commonly observed^10,18^. Our findings may not generalize to settings with lower loss rates or stronger free-riding incentives. Future research would be valuable in examining how background risk affects cooperation under less favorable coordination conditions. Second, following previous studies^15,17,31^, contributions to the climate fund in the PGG are linked to real-world CO_2_ emission reductions to enhance realism, while also ensuring comparability with related experiments. However, this design feature may have introduced an additional utility component associated with contributing. Participants may have perceived their contributions not only in terms of reaching the threshold within the experimental task, but also as an opportunity to generate meaningful environmental benefits in real life. The high provision rates observed across all control and treatment conditions could partially reflect this consideration. It is worth exploring in future research how willingness to cooperate in climate action is shaped more generally, and how this willingness may further change under background risk when real-world consequences are not involved. Finally, we implemented a fixed 20% background risk. In reality, however, climate protection is a long-term process extending over years or even decades. Background risk is unlikely to remain constant; instead, it may vary over time, becoming more salient or pronounced during certain periods and less so during others. Individuals may also experience background risk heterogeneously, facing different types and levels of external uncertainties. As a result, behavioral responses to background risk may adapt dynamically over time. This dynamic and heterogeneous nature of background risk is not captured in the present experimental design and may offer a valuable avenue for future research to better reflect real-world conditions. Further research is needed to assess the generalizability of the observed effects of background risk.

Our experimental results suggest that when losses and damages from climate change are salient and well-communicated, background risk does not substantially weaken cooperative behavior. Under such favorable coordination environments, concerns that background risk might undermine climate cooperation may be less pronounced than predicted by theory. This highlights that the benefits of transparent and clear climate communication may be twofold: when the public is aware of the potential consequences of climate change, willingness to cooperate remains generally high; moreover, under such conditions, the theoretically expected reduction in cooperation due to background risk appears less relevant. Future climate communication strategies may benefit from considering this aspect.

## Supplementary Information

Supplementary Information.

## Data Availability

The data and code used in the paper are available at OSF repository and can be accessed at: https://osf.io/9tkxa/?view_only=9af9f1a588ee4822b02d34cfe91d2325.
