# Supplementary material for: Cooperative climate action under background risk
Source: Sci Rep. 2025 Jul 25;15:27118. doi: 10.1038/s41598-025-12340-9 (PMC12297547; doi:10.1038/s41598-025-12340-9)
Supplement: Supplementary file 1 — Supplementary Information. [file 41598_2025_12340_MOESM1_ESM.pdf]

Supplementary Information for
Cooperative climate action under background risk

Hao Luo*[, a], Hanna de Boer[b], Oliver Musshoff[a], and Daniel Hermann[b]

[a] Department of Agricultural Economics and Rural Development, University of Göttingen, Göttingen D-37073, Germany
[b] Institute for Food and Resource Economics, University of Bonn, Bonn D-53115, Germany

[*] Corresponding author. Email: hao.luo@uni-goettingen.de

This PDF file includes:

Supplementary Note 1
Figure S1

Supplementary Note 1. The experimental instruction (English translation).

Control Group

Dear Participant,

Welcome to our experiment!

During the experiment, it is not permitted to communicate with other participants in any form. If you have any questions, please raise your hand to ask us directly. Additionally, using your mobile phone or listening to music is not allowed.

In this experiment, you can earn a varying amount of money depending on your decisions. Your earnings will be expressed in tokens, which will be converted into euros at the following exchange rate at the end of the experiment:

1 Token = 0.25 Euros

Additionally, you will receive a guaranteed participation fee of **10 Euros** for taking part. Your total earnings will be paid to you in cash after the experiment, without other participants knowing how much you earned.

The experiment will last approximately **60 minutes** and consists of two games and a few questions. It is important that you carefully read the instructions you receive. Your anonymity is guaranteed throughout the experiment, and it will not be possible to trace your personal responses back to the collected data.

By clicking "Next," you agree to participate in this experiment.

Thank you for taking part in our experiment!

In the following game, the computer will randomly assign you to a group of **6 participants**. Within your group, you will be randomly assigned a position as **Player 1, Player 2, Player 3, Player 4, Player 5, or Player 6**. This position will be referred to as your **"position."** You will remain in the same group throughout the game, and your position within the group will not change. The identities of the participants in each group will remain confidential both during and after the experiment.

Comprehension Questions

1. How many participants are in your group?
   Answer: 6
2. Do positions within the group change during the game?
   Answer: No [Checkbox (Yes [], No [X])]

The game consists of **10 periods**. In each period, you must make a decision.

At the start of each period, you will receive **4 tokens**, which you can allocate between a **private account** and a **public climate fund**. The tokens in the climate fund are used to combat the causes and effects of climate change and are **not distributed among group members**. You can only invest **0, 2, or 4 tokens** in the climate fund.

The contributions of all **6 participants** in your group will be summed up in the climate fund. By the end of the game (**after 10 periods**), the total contribution in the climate fund must reach **120 tokens** to provide sufficient climate protection.

- If the group **reaches 120 tokens** in the climate fund, all participants **keep** the remaining balance in their private accounts.
- If the group **does not reach 120 tokens** in the climate fund, each participant **receives only 10%** of their remaining private account balance.

Starting from the **second period**, you will receive information after each decision about the contributions of the other participants as well as the total group contributions from the previous round. Additionally, you will see the cumulative contributions of each player and the entire group. To ensure anonymity, participants are referred to as **Player 1 to Player 6**.

At the end of the game, tokens in the climate fund will be converted into euros and used to **purchase EU CO_2 certificates**. **If the group reaches 120 tokens**, **100% of the climate fund money** will be used for certificate purchases. **If the group does not reach 120 tokens**, only **50% of the climate fund money** will be used.

CO_2 certificates are **tradable permits** that allow companies to emit a certain amount of CO_2. When these certificates are bought and removed from circulation, the total number of emission rights decreases, leading to a **long-term reduction in CO_2 emissions**. Thus, your contributions also contribute to **real-life climate protection**.

The purchased certificates will be published after the experiment on the **website of the Research Group for Digital Transformation and Circular Economy at the Institute for Food and Resource Economics**.

Comprehension Questions

3. How much must each participant contribute per period, on average, to reach a total investment of 120 tokens?
   Answer: 2
4. Assume your group reaches the minimum threshold of 120 tokens after 10 periods, and you have contributed a total of 16 tokens to the climate fund. How many tokens will you receive at the end of the game?
   Answer: 24
5. Assume you have contributed a total of 20 tokens to the climate fund, but the minimum threshold of 120 tokens was not reached. What percentage of your private account balance will you receive at the end of the game?
   Answer: 10%

Correct!

By clicking "Start," the game will begin.

Good luck!

The computer has randomly assigned your group and your position within the group.
Your position in the group is: **Player 1**

Treatment 1

Dear Participant,

Welcome to our experiment!

During the experiment, it is not permitted to communicate with other participants in any form. If you have any questions, please raise your hand to ask us directly. Additionally, using your mobile phone or listening to music is not allowed.

In this experiment, you can earn a varying amount of money depending on your decisions. Your earnings will be expressed in tokens, which will be converted into euros at the following exchange rate at the end of the experiment:

1 Token = 0.25 Euros

Additionally, you will receive a guaranteed participation fee of **10 Euros** for taking part. Your total earnings will be paid to you in cash after the experiment, without other participants knowing how much you earned.

The experiment will last approximately **60 minutes** and consists of two games and a few questions. It is important that you carefully read the instructions you receive. Your anonymity is guaranteed throughout the experiment, and it will not be possible to trace your personal responses back to the collected data.

By clicking "Next," you agree to participate in this experiment.

Thank you for taking part in our experiment!

In the following game, the computer will randomly assign you to a group of **6 participants**. Within your group, you will be randomly assigned a position as **Player 1, Player 2, Player 3, Player 4, Player 5, or Player 6**. This position will be referred to as your **"position."** You will remain in the same group throughout the game, and your position within the group will not change. The identities of the participants in each group will remain confidential both during and after the experiment.

Comprehension Questions

1. How many participants are in your group?
   Answer: 6
2. Do positions within the group change during the game?
   Answer: No [Checkbox (Yes [], No [X])]

The game consists of **10 periods. I**n each period, you must make a decision.

At the start of each period, you will receive **4 tokens**, which you can allocate between a **private account** and a **public climate fund**. The tokens in the climate fund are used to combat the causes and effects of climate change and are **not distributed among group members**. You can only invest **0, 2, or 4 tokens** in the climate fund.

The contributions of all **6 participants** in your group will be summed up in the climate fund. By the end of the game (**after 10 periods**), the total contribution in the climate fund must reach **120 tokens** to provide sufficient climate protection.

- If the group **reaches a total of 120 tokens** in the climate fund, each of the 6 participants has **a 20% probability of losing their entire private account balance**.
- If the group **does not reach 120 tokens** in the climate fund, each participant **receives only 10% of their private account balance**, and there is also **a 20% probability that this remaining balance will be lost entirely**.

Starting from the **second period**, you will receive information after each decision about the contributions of the other participants as well as the total group contributions from the previous round. Additionally, you will see the cumulative contributions of each player and the entire group. To ensure anonymity, participants are referred to as **Player 1 to Player 6**.

At the end of the game, tokens in the climate fund will be converted into euros and used to **purchase EU CO_2 certificates**. **If the group reaches 120 tokens**, **100% of the climate fund money** will be used for certificate purchases. **If the group does not reach 120 tokens**, only **50% of the climate fund money** will be used.

CO_2 certificates are **tradable permits** that allow companies to emit a certain amount of CO_2. When these certificates are bought and removed from circulation, the total number of emission rights decreases, leading to a **long-term reduction in CO_2 emissions**. Thus, your contributions also contribute to **real-life climate protection**.

The purchased certificates will be published after the experiment on the **website of the Research Group for Digital Transformation and Circular Economy at the Institute for Food and Resource Economics**.

Comprehension Questions

3. How much must each participant contribute per round, on average, to reach a total investment of 120 tokens?
   Answer: 2

4. If you have contributed 20 tokens to the climate fund and the group has reached the minimum threshold of 120 tokens, what is the probability (%) that you will keep all tokens in your private account at the end of the game?
   Answer: 80%

5. If you have contributed 20 tokens to the climate fund and the group has not reached the minimum threshold of 120 tokens, what is the probability (%) that you will keep your remaining private account balance at the end of the game?

Answer: 80%

Correct!

By clicking "Start," the game will begin.

Good luck!

The computer has randomly assigned your group and your position within the group.
Your position in the group is: **Player 1**

Treatment 2

Dear Participant,

Welcome to our experiment!

During the experiment, it is not permitted to communicate with other participants in any form. If you have any questions, please raise your hand to ask us directly. Additionally, using your mobile phone or listening to music is not allowed.

In this experiment, you can earn a varying amount of money depending on your decisions. Your earnings will be expressed in tokens, which will be converted into euros at the following exchange rate at the end of the experiment:

1 Token = 0.25 Euros

Additionally, you will receive a guaranteed participation fee of **10 Euros** for taking part. Your total earnings will be paid to you in cash after the experiment, without other participants knowing how much you earned.

The experiment will last approximately **60 minutes** and consists of two games and a few questions. It is important that you carefully read the instructions you receive. Your anonymity is guaranteed throughout the experiment, and it will not be possible to trace your personal responses back to the collected data.

By clicking "Next," you agree to participate in this experiment.

Thank you for taking part in our experiment!

In the following game, the computer will randomly assign you to a group of **6 participants**. Within your group, you will be randomly assigned a position as **Player 1, Player 2, Player 3, Player 4, Player 5, or Player 6**. This position will be referred to as your **"position."** You will remain in the same group throughout the game, and your position within the group will not change. The identities of the participants in each group will remain confidential both during and after the experiment.

Comprehension Questions

1. How many participants are in your group?
   Answer: 6
2. Do positions within the group change during the game?
   Answer: No [Checkbox (Yes [], No [X])]

The game consists of **10 periods**. In each period, you must make a decision.

At the start of each period, you will receive **4 tokens**, which you can allocate between a **private account** and a **public climate fund**. The tokens in the climate fund are used to combat the causes and effects of climate change and are **not distributed among group members**. You can only invest **0, 2, or 4 tokens** in the climate fund.

The contributions of all **6 participants** in your group will be summed up in the climate fund. By the end of the game (**after 10 periods**), the total contribution in the climate fund must reach **120 tokens** to provide sufficient climate protection.

- If the group **reaches a total of 120 tokens** in the climate fund, the amount in each private account is **first multiplied by 1.25**. After that, each of the 6 participants has a **20% probability of losing their entire multiplied private account balance**.
- If the group **does not reach 120 tokens** in the climate fund, the amount in each private account is also **multiplied by 1.25**. However, after this multiplication, each participant receives **only 10% of the new balance**, and there is a **20% probability of losing this remaining amount entirely**.

Starting from the **second period**, you will receive information after each decision about the contributions of the other participants as well as the total group contributions from the previous round. Additionally, you will see the cumulative contributions of each player and the entire group. To ensure anonymity, participants are referred to as **Player 1 to Player 6**.

At the end of the game, tokens in the climate fund will be converted into euros and used to **purchase EU CO_2 certificates**. **If the group reaches 120 tokens**, **100% of the climate fund money** will be used for certificate purchases. **If the group does not reach 120 tokens**, only **50% of the climate fund money** will be used.

CO_2 certificates are **tradable permits** that allow companies to emit a certain amount of CO_2. When these certificates are bought and removed from circulation, the total number of emission rights decreases, leading to a **long-term reduction in CO_2 emissions**. Thus, your contributions also contribute to **real-life climate protection**.

The purchased certificates will be published after the experiment on the **website of the Research Group for Digital Transformation and Circular Economy at the Institute for Food and Resource Economics**.

Comprehension Questions

3. How much must each participant contribute per round, on average, to reach a total investment of 120 tokens?
   Answer: 2

4. If you have contributed 20 tokens to the climate fund and the group has reached the minimum threshold of 120 tokens, what is the probability (%) that you will keep all tokens in your private account at the end of the game?
   Answer: 80%

5. If you have contributed 20 tokens to the climate fund and the group has reached the minimum threshold of 120 tokens, how much will you have in your private account after multiplication but before the lottery?
   Answer: 25

Correct!

By clicking "Start," the game will begin.

Good luck!

The computer has randomly assigned your group and your position within the group.
Your position in the group is: **Player 1**

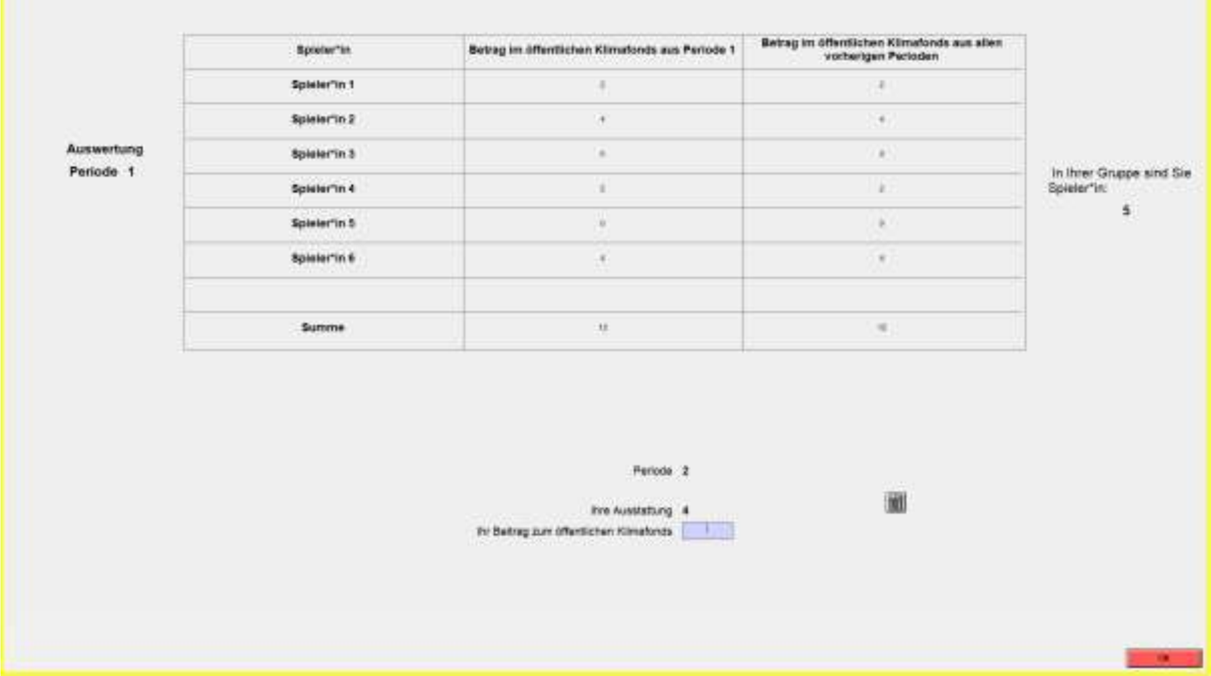

Figure S1. Example of the game interface in period 2

Ex-post questionnaire

1. Age: Please indicate your age in years

2. Gender
   Please indicate your gender identity by selecting one of the circles:
   a) Female;

b) Male;
c) Diverse

3. To which faculty does your field of study belong? Please select one of the circles or use the open response field below.
   a) Faculty of Protestant Theology
   b) Faculty of Catholic Theology
   c) Faculty of Agricultural, Nutritional, and Engineering Sciences
   d) Faculty of Mathematics and Natural Sciences
   e) Faculty of Medicine
   f) Faculty of Philosophy
   g) Faculty of Law and Political Science
   h) Other (please specify below)

4. Please indicate which religious community you belong to by selecting one of the circles or using the open response field below.
   a) Christianity
   b) Judaism
   c) Islam
   d) Hinduism
   e) Buddhism
   f) None
   g) Other (please specify below)

5. Many people use the terms "left" and "right" when they want to describe different political views. Here we have a scale which runs from left to right. Thinking of your own political views, where would you place these on this scale? Please select one of the circles.

6. How would you rate your current income on a scale from 1 to 10, where 1 represents very low income and 10 represents extremely high income? Please select one circle.

7. How well do the following statements describe your personality? The rating scale ranges from "does not apply at all" (1) to "fully applies" (5). Please select one circle for each statement.
   a) I see myself as someone who is reserved.
   b) I see myself as someone who is generally trusting.
   c) I see myself as someone who tends to be lazy.
   d) I see myself as someone who is relaxed, handles stress well.
   e) I see myself as someone who has few artistic interests.
   f) I see myself as someone who is outgoing, sociable.
   g) I see myself as someone who tends to find fault with others.
   h) I see myself as someone who does a thorough job.
   i) I see myself as someone who gets nervous easily.
   j) I see myself as someone who has an active imagination.

8. Below you will find 10 alternatives, each with 2 lottery options. Your task is to choose one of the two lottery options in each row based on your preference. Depending on your choice and the lottery outcomes, you can earn tokens. For the payment of this task, one decision will be randomly selected, and its outcome will be paid out. Please indicate your selection by choosing one circle for each decision situation.

	Option A	Option B	Your choice
1	10% of 8.00 tokens; 90% of 6.40 tokens	10% of 15.40 tokens; 90% of 0.40 tokens	A O B
2	20% of 8.00 tokens; 80% of 6.40 tokens	20% of 15.40 tokens; 80% of 0.40 tokens	A O B
3	30% of 8.00 tokens; 70% of 6.40 tokens	30% of 15.40 tokens; 70% of 0.40 tokens	A O B
4	40% of 8.00 tokens; 60% of 6.40 tokens	40% of 15.40 tokens; 60% of 0.40 tokens	A O B
5	50% of 8.00 tokens; 50% of 6.40 tokens	50% of 15.40 tokens; 50% of 0.40 tokens	A O B
6	60% of 8.00 tokens; 40% of 6.40 tokens	60% of 15.40 tokens; 40% of 0.40 tokens	A O B
7	70% of 8.00 tokens; 30% of 6.40 tokens	70% of 15.40 tokens; 30% of 0.40 tokens	A O B
8	80% of 8.00 tokens; 20% of 6.40 tokens	80% of 15.40 tokens; 20% of 0.40 tokens	A O B
9	90% of 8.00 tokens; 10% of 6.40 tokens	90% of 15.40 tokens; 10% of 0.40 tokens	A O B
10	100% of 8.00 tokens; 0% of 6.40 tokens	100% of 15.40 tokens; 0% of 0.40 tokens	A O B

9. Please indicate the extent to which you agree with the following statements (1 = strongly disagree, 5 = strongly agree). Please select one circle for each statement.
   a) Climate change is occurring, and it is caused mostly by human activities.
   b) Climate change is occurring, and it is caused more or less equally by natural changes in the environment and human activities.
   c) Climate change is occurring, and it is caused mostly by natural changes in the environment.
   d) There is not sufficient evidence to know with certainty whether climate change is occurring or not. Climate change is not occurring.
